# Impacts of Sub-lethal DDT Exposures on microRNA and Putative Target Transcript Expression in DDT Resistant and Susceptible *Drosophila melanogaster* Strains

**DOI:** 10.3389/fgene.2019.00045

**Published:** 2019-02-05

**Authors:** Keon Mook Seong, Brad S. Coates, Barry R. Pittendrigh

**Affiliations:** ^1^Department of Entomology, Michigan State University, East Lansing, MI, United States; ^2^USDA-ARS, Corn Insects and Crop Genetics Research Unit, Ames, IA, United States

**Keywords:** *Drosophila melanogaster*, microRNA, DDT resistance, expression analysis, cytochrome P450

## Abstract

Ten constitutively differentially expressed miRNAs were previously described between DDT-resistant *91-R* and -susceptible control *Drosophila melanogaster* strains, and among their predicted target genes were those associated with metabolic DDT resistance mechanisms. The present study evaluated the inducibility of miRNA expression and putative downstream regulation of cytochrome P450s in response to DDT exposure in a time-dependent manner in *91-R* and the susceptible *Canton-S* strain. Specifically, RT-qPCR analysis showed that DDT exposures led to the significant down-regulation (repression) of *miR-310-3p*, *miR-311-3p*, *miR-312-3p*, *miR-313-3p*, and *miR-92a-3p* levels in *Canton-S*. This is contrasted with the lack of significant changes in *91-R* at most time-points following DDT exposure. The levels of expression among miRNAs exhibited opposite expression patterns compared to their corresponding putative target cytochrome P450s at the same time points after DDT exposure. Collectively, results from this study suggest that *miR-310-3p*, *miR-311-3p*, *miR-312-3p*, *miR-313-3p*, and *miR-92a-3p* might have a potential role in the control of DDT detoxification through the post-transcriptional regulation of target cytochrome P450s in *Canton-S*. Conversely, the lack of significant changes of these same miRNAs in *91-R* following DDT-exposure suggests a possible adaptive mutation that removes repressive control mechanisms. These data are important for the understanding impact of adaptive changes in miRNA expression on post-transcriptional regulatory mechanism involved in the evolution of DDT resistance in *91-R*.

## Introduction

For well over half a century, second generation insecticides have been used across the globe as one of the most cost-effective ways to control pest insects, directly impacting human health through methods of crop production and the management of vector-borne diseases. However, the extensive and frequent application of insecticide has contributed to the evolution of insect populations with resistance to one or more classes of insecticides (Mallet, 1989). The organochlorine insecticide dichlorodiphenyltrichloroethane (DDT) was widely used in the decades post-WWII for the control of agricultural and disease vectoring pest insects (Edman, 2004; Hemingway, 2009), however, it was ultimately banned in most countries due to deleterious environmental side effects as well as its impacts on non-target mammalian and avian species (Krupke et al., 2007). Although DDT is no longer used for agricultural pest control, DDT resistance in the fruit fly, *Drosophila melanogaster* (hereafter referred to as *Drosophila*), provides a model system for studying the evolution of insecticide resistance (Wilson, 1988). In some *Drosophila* strains, historically, moderate and high levels of DDT resistance have been considered to be polygenic and associated with contribution of multiple resistance genes to the phenotype (Kim et al., 2018), including metabolic-based resistance influenced by the alteration of enzyme activities that degrade or sequester insecticides (Li et al., 2007). Metabolic resistance to DDT in *Drosophila* has been associated with constitutively up-regulated genes and increased quantities of cytochrome P450s (P450s), glutathione-*S*-transferases (GSTs), and esterases (Feyereisen, 1999; Vontas et al., 2002; Tu and Akgul, 2005). DDT resistance in *Drosophila* also is dependent upon expression of a Phase III detoxification gene, the ABC transporter, which likely may enhance excretion of xenobiotic metabolites through the up-regulation of *mdr50* and *mdr65* (Strycharz et al., 2013) and the structural change of *mdr49* in DDT resistant phenotypes (Seong et al., 2016). Furthermore, DDT resistance in the *Drosophila* strain *91-R* has been associated with genetic and signal transduction pathways that converge to modify stress response, cell survival, and neurological functions (Seong et al., 2017).

A large number of studies have focused on the role of P450s in the detoxification of xenobiotic compounds in insect species. Much of the DDT resistance observed in *Drosophila* has been attributed to constitutive increases in expression of the P450s *Cyp6g1*, *Cyp6a2*, and *Cyp12d1* (Festucci-Buselli et al., 2005; Kuruganti et al., 2007; Jones et al., 2010), and amino acid changes in CYP6W1 involved in the metabolism of DDT resistance phenotype (Schmidt et al., 2010). Seong et al. (2018a) recently described the evolutionary adaptive response of functional and structural variation within the P450 gene family as a result of chronic high levels of DDT exposure in the DDT-resistant strain *91-R* when compared to its control strain, *91-C*. Additionally, the transcriptional regulation of P450s expression has become of the focus of recent studies to better understand the underlying mechanisms involved in constitutive over-transcription of P450 genes in pesticide resistant insects. For example, the Nrf2/Maf transcription factor-mediated regulatory pathway governing *Cyp6a2* gene up-regulation is associated with DDT resistance in *Drosophila* (Wan et al., 2014). More recent studies have demonstrated the functional roles of microRNAs (miRNAs) in regulating the expression of P450s that are in turn involved in insecticide resistance in insect species (Hong et al., 2014; Etebari et al., 2018).

The miRNAs are endogenous small non-coding RNA species that are generally 19–25 nucleotides in length, and function in the post-transcriptional regulation of gene expression by directly targeting to the 3′ untranslated region (3′ UTR) of mRNA targets (Bartel, 2004). miRNAs can play important regulatory roles in biological processes including development, immunity, and metamorphosis (Ambros, 2004; Wei et al., 2018). Several studies have characterized the role of miRNAs in responses to environmental chemical and pathogen exposures. For instance, a number of miRNAs are differentially expressed in a dose-dependent response to copper concentrations in zebrafish and these up/down-regulated miRNAs were predicted to post-transcriptionally control of transcripts involved in olfactory signal transduction pathways and neurological processes (Wang et al., 2013). miRNAs also have complex interactions with *Drosophila* innate immune systems in response to bacterial infections (Wei et al., 2018), which can lead to dynamic changes in miRNA expression and downstream regulation of multiple mRNAs targets involved in *Drosophila* immune response. Strikingly, miRNAs have attracted considerable attention for their involvement in mechanisms of resistance to insecticides such as DDT (Seong et al., 2018b), permethrin (pyrethroid) (Ma et al., 2017), fenpropathrin (Zhang et al., 2016), and imidacloprid (Morin et al., 2017). A number of studies have shown that several miRNAs are significantly differentially-expressed in insects following exposure to different insecticides, and a subset of their putative target genes are involved metabolic detoxification processes. For example, quantitative comparison of miRNA libraries from chlorantraniliprole-resistant and -susceptible strains of *Plutella xylostella* revealed that a number of miRNAs were differentially expressed and their predicted target genes were associated with chlorantraniliprole resistance mechanisms (Zhu et al., 2017). Furthermore, *miR-2b-3p* was predicted to regulate the expression of a putative P450 target gene, *Cyp9f2*, in *P. xylostella* (Etebari et al., 2018). In a recent study, levels of *Cyp9j5* and *Cyp325bg3* transcripts in *Culex pipiens* were significantly decreased upon *miR-2∼13∼71* cluster mimic injection, which demonstrated the regulation of these target genes by a regulatory miRNA (Guo et al., 2017). Moreover, *miR-1-3p* may be involved in cyflumetofen resistance by targeting an mRNA encoding a GST in *Tetranychus cinnabarinus* (Zhang et al., 2018).

A number of studies suggest that the induction of detoxification genes, such as P450s, play a critical role in the metabolism of insecticide in insects and may also be adaptive (Liu et al., 2011; Gong et al., 2013). However, the role of insecticide induced miRNA expression in the post-transcriptional regulation of genes associated with insecticide resistance in *Drosophila* remains poorly understood. Our previous studies comparing constitutive miRNA expression levels between DDT-resistant and -susceptible *Drosophila* strains identified multiple up- and down-regulated miRNAs predicted to potentially contribute for the development of DDT resistance via targeting transcripts of multiple detoxification genes. Conversely, there has been no corresponding studies demonstrating any inducible miRNA response to pesticide exposure in pesticide resistant and susceptible strains. In the present study, the induction or repression of miRNAs in response to DDT exposure is demonstrated in the DDT-resistant *91-R* and -susceptible *Canton-S Drosophila* strains and are putatively linked to the regulation of transcript levels of their target P450 genes. The study also reports that, when compared to their susceptible counterpart, the DDT resistance phenotype in *91-R* is associated with constitutively lower levels of the same miRNAs. These data are important for understanding the potential roles of differential miRNA expression on the evolution of DDT resistance in the *Drosophila* strains, and may provide insight into processes that lead to the evolution of phenotypes adapted to increased survivorship when exposed to xenobiotics.

## Materials and Methods

### *Drosophila* Strains

For the purposes of this study, two *Drosophila* strains, *91-R* and *Canton-S*, were reared and used as previously described (Seong et al., 2017). The *91-R* strain has remained under continual selection by maintaining the flies in bottles with filter paper disks impregnated with 150 mg/ml of DDT, while *Canton-S* has been maintained without any exposure to insecticides. The *91-R* strain was previously shown to be 1,526-fold more resistant to DDT compared to the *Canton-S* susceptible strain using contact vial bioassays (Strycharz et al., 2013).

### DDT Treatment

To determine *Drosophila* miRNA responses to DDT, the response of miRNA expression was determined by using DDT contact exposure bioassays as described previously (Seong et al., 2016). In brief, 15 3-day-old female adult flies from each the *91-R* and *Canton-S* strains were exposed to vials coated with DDT at their respective LC_10_ (5 mg/vial for *91-R*; 1.5 μg/vial for *Canton-S*), and samples taken at 12, 24, 48, and 72 h after the DDT exposure for each strain. Control flies that had not received the DDT treatment (treated with acetone alone) were collected at the same time points as DDT treatment counterparts. Each treatment by time point experiment was performed in triplicate.

### RNA Extraction and cDNA Preparation

Total RNA was extracted per replicate from a pool of 15 flies surviving DDT treatment and acetone treatment using the Qiagen miRNeasy Mini Kit according to the manufacturer’s instructions (Qiagen, Valencia, CA, United States), which was followed by treatment of each sample with DNase I (Qiagen) before cDNA synthesis to remove contaminating genomic DNA. First-strand cDNA was synthesized using the miScript II RT kit (Qiagen) with the provided miScript HiFlex Buffer according to the manufacturer’s instructions.

### Gene Expression by Reverse Transcriptase-Quantitative PCR (RT-qPCR)

RT-qPCR validation was performed on eight miRNAs (*miR-310-3p*, *miR-311-3p*, *miR-312-3p*, *miR-313-3p*, *miR-92a-3p*, *miR-286-3p*, *miR-4919-3p*, and *miR-986-5p)*, which were estimated to be constitutively differentially expressed between DDT-resistant *91-R* and -susceptible *91-C* strains by Seong et al. (2018b). RT-qPCR reactions were performed using the miScript SYBR^®^ Green PCR Kit (Qiagen) according to the manufacturer’s instructions with miRNA-specific forward primers and target mRNA-specific primers (Supplementary Table S1). All RT-qPCR reactions were carried out on a StepOnePlus Real-Time PCR system (Applied Biosystems, Inc., Foster City, CA, United States), with three technical replicates across all biological replicates. Normalized miRNA and target transcript expression levels were calculated using the 2^-ΔΔC(t)^ method (Schmittgen and Livak, 2008) with the references *U6 snRNA* and *5S rRNA* for miRNA normalization and *rp49* for target mRNA normalization. The amplification efficiency of target miRNAs, mRNAs and reference genes were estimated using the formula: E = 10(-1/slope) where slope was derived from plotting the cycle threshold (Ct) value versus five serially 10^n^-fold diluted cDNA template concentrations. RT-qPCR was performed across three biological replicates. The statistical analysis of gene expression was performed using a two-way analysis of variance (ANOVA) to determine the changes in miRNA expression level through DDT exposure is dependent on the genetic background with Tukey’s multiple comparison test (XLSTAT 2008, Addinsoft, United States); a *P*-value < 0.05 was considered statistically significant. Pearson correlation coefficients between a particular miRNA and its predicted target P450 mRNAs were computed to determine whether the expression levels of each miRNA and of its mRNA targets were negatively correlated. Correlation were considered significant at *P* < 0.05.

### Potential Target P450s Prediction of Eight Differentially Expressed miRNAs

The putative P450 target transcripts of miRNAs investigated in this study were previously predicted using three different types of software packages (RNAhybrid, DIANA, and ComiR) as described by Seong et al. (2018b) and another software, miRanda, to predict potential target P450s (Betel et al., 2010). In brief, for each prediction method, high efficacy targets were selected by the following criteria: (1) RNAhybrid: the target site prediction was restricted to the 3′-UTR region obtained from the 3′-UTR database of *D. melanogaster* (dmel-all-three_prime_UTR-r6.19.fasta at http://www.flybase.org) with MFE ≤-30 kcal mol^-1^; (2) DIANA: miTG score ≥ 0.8; (3) ComiR: high threshold ≥ 0.8; (4) miRanda: total score ≥ 120, total energy ≤-25 kcal/mol.

## Results

### Time-Dependent Expression of Multiple miRNAs in Response to DDT Exposure in DDT-Resistant *91-R* and -Susceptible *Canton-S* Strains

Among 10 miRNAs previously shown to be differentially expressed between *91-R* and *91-C* strains (Seong et al., 2018b), eight miRNAs (*miR-310-3p*, *miR-311-3p*, *miR-312-3p*, *miR-313-3p*, *miR-92a-3p*, *miR-286-3p*, *miR-4919-3p*, and *miR-986-5p*) showed significant levels of differential expression and were herein investigated with respect to induction or repression in DDT resistant *91-R* and DDT susceptible *Canton-S* strains following DDT exposure. No significant changes were detected in miRNA expression (induction or repression) for any of the eight miRNAs within acetone-treated control flies from either strain across any sampling time points (*P*-values ≥ 0.05; Supplementary Table S2). In contrast, five miRNAs (*miR-310-3p*, *miR-311-3p*, *miR-312-3p*, *miR-313-3p*, *miR-92a-3p*) were significantly down-regulated following DDT exposure in *Canton-S* strain as compared to acetone treated controls. Repression of these miRNAs occurred universally across 12 to 72 h after DDT exposure (*P-*values < 0.05).

**FIGURE 1:**
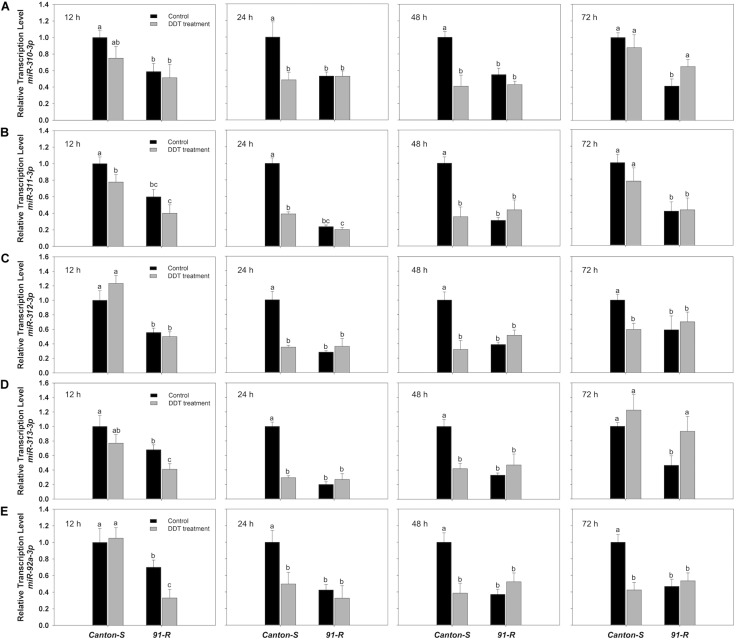
The expression of miR-310 family, **(A)** *miR-310*, **(B)** *miR-311*, **(C)** *miR-312*, **(D)** *miR-313*, and **(E)** *miR-92a*, in DDT-resistant *91-R* and the DDT-susceptible *Canton-S* strains following DDT exposure. The induction or repression level of expression were analyzed 12, 24, 48, and 72 h after DDT exposure. The relative levels of gene expression shown along the Y axis represent the ratio of the gene expression in each treatment compared to the acetone-treated *Canton-S* strain. The experiments were repeated three times. The vertical bars indicate standard error of the mean (SEM). Different letters on the bars indicate that the means are significantly different throughout the strains where the *P*-value < 0.05.

**FIGURE 2:**
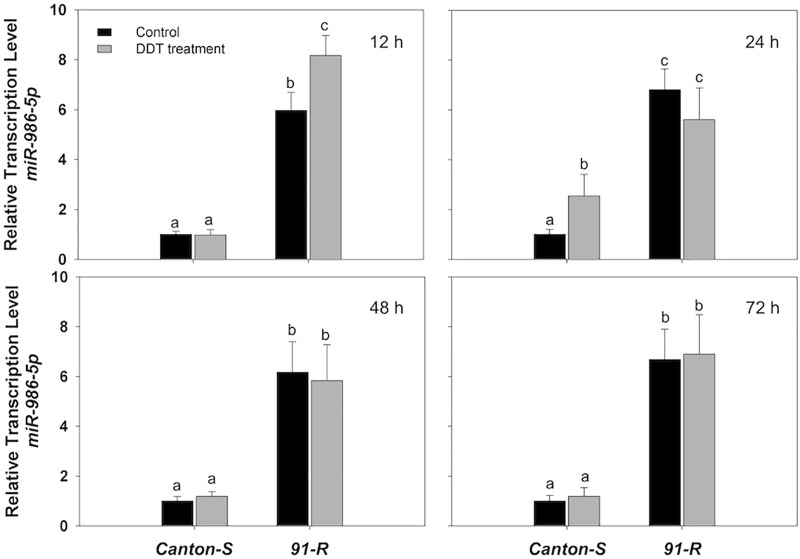
The expression of *miR-986-5p* in DDT-resistant *91-R* and the DDT-susceptible *Canton-S* strains following DDT exposure. The induction or repression level of expression were analyzed 12, 24, 48, and 72 h after DDT exposure. The relative levels of gene expression shown along the Y axis represent the ratio of the gene expression in each treatment compared to the acetone-treated *Canton-S* strain. The experiments were repeated three times. The vertical bars indicate SEM. Different letters on the bars indicate that the means are significantly different throughout the strains where the *P*-value < 0.05.

Specifically, the initial repression of *miR-311-3p* in *Canton-S* occurred at 12 h (0.78-fold), followed by a 0.39- and 0.35-fold decreases in expression respectively at 24 and 48 h after DDT treatment compared to the acetone-treated control flies (Figure 1B). Similarly, the initial down-regulation of *miR-310-3p*, *miR-312-3p*, *miR-313-3p*, and *miR-92a-3p* occurred at 24 h after exposure to DDT with respective down-regulation of 0.48-, 0.35-, 0.29-, and 0.5-fold as compared to the control flies (Figure 1A,C–E). In the *Canton-S* strain, the expression of *miR-310-3p*, *miR-311-3p*, *miR-312-3p*, and *miR-92a-3p* were most highly down-regulated at 48 h after exposure to DDT with reduced levels respectively of 0.41-, 0.35-, 0.32-, and 0.39-fold (Figure 1A–C,E). Comparatively, *miR-313-3p* was most highly down-regulated in *Canton-S* at 24 h after DDT treatment with a reduced level of 0.29-fold (Figure 1D). At 72 h after DDT exposure, the expression of *miR-310-3p*, *miR-311-3p*, and *miR-313-3p* showed no significant difference compared with acetone-treated flies (Figure 1A,B,D), whereas the *miR-312-3p* and *miR-92a-3p* remained down-regulated after 72 h post-DDT treatment in *Canton-S* with approximate 0.6- and 0.42-fold reductions (Figure 1C,E). In contrast, the estimated expression of *miR-986-5p* increased by approximately 2.6-fold at 24 h after DDT treatment in *Canton-S* strain (Figure 2). Both *miR-286-3p* and *miR-4919-3p* showed no significant difference at any time points after DDT treatment as compared to the acetone-treated control (*P*-values ≥ 0.05) in both *91-R* and *Canton-S* strains (Supplementary Figures S1, S2).

The miRNAs in *91-R* were constitutively expressed and for the most part showed non-significant degrees of change in response to DDT exposure. Specifically, analysis of RT-qPCR results indicated that all eight miRNAs showed significant levels of differential expression between *91-R* and *Canton-S* across time points post-DDT exposure, whereby seven miRNAs were significantly down-regulated, and only one (*miR-986-5p*) was significantly up-regulated in *91-R* as compared to *Canton-S* (*P*-values < 0.05). DDT exposure induced the up-regulation of two miRNAs (*miR-310-3p* and *miR-313-3p*) with varying levels and in a time-dependent manner in *91-R* strain. The expression of *miR-310-3p* in *91-R* strain reached maximum level of induction (1.59-fold) at 72 h after exposure to DDT (Figure 1A). Similarly for *miR-313-3p*, the significant induction level was also observed at 72 h with maximum expression levels of 2.0-fold (Figure 1D). Similar to *Canton-S* strain, down-regulation of miRNAs by DDT exposure was shown for *miR-313-3p*, and *miR-92a-3p* in *91-R* strain only at 12 h after treatment of DDT with expression level reduced 0.6- and 0.42-fold, respectively, as compared to the acetone-treated control (Figure 1D,E). For both *miR-286-3p* and *miR-4919-3p*, no inducibility of expression was detected in *91-R* tested at any time points after treatment with DDT at LC_10_ (Supplementary Figures S1, S2). In the *91-R* strain, the initial induction of up-regulation resulting in a 1.37-fold change in of *miR-986-5p* occurred at 12 h after exposure to DDT, but no significant induction was found after 24 h following DDT treatment (Figure 2). Overall, the expression level of miRNAs in *91-R* showed no significant difference at most time points after DDT treatment compared to the acetone-treated control (*P*-values ≥ 0.05). Notably, the two-way treatment × genotype statistical interaction showed that the differentiation of most miRNA expression induced by DDT exposure at each time point is dependent on the genetic background of fly strains (*P-*values < 0.05; Supplementary Table S3).

### Co-expression Patterns Between miRNAs and Their Potential Target P450 mRNAs

**FIGURE 3:**
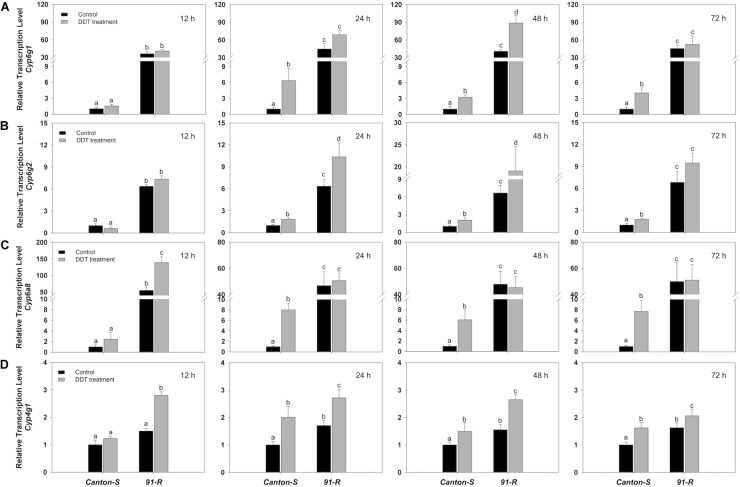
The expression of target P450s **(A)** *Cyp6g1*, **(B)** *Cyp6g2*, **(C)** *Cyp6a8*, and **(D)** *Cyp4g1* in DDT-resistant *91-R* and the DDT-susceptible *Canton-S* strains following DDT exposure. The induction or repression level of expression were analyzed 12, 24, 48, and 72 h after DDT exposure. The relative levels of gene expression shown along the Y axis represent the ratio of the gene expression in each treatment compared to the acetone-treated *Canton-S* strain. The experiments were repeated three times. The vertical bars indicate SEM. Different letters on the bars indicate that the means are significantly different throughout the strains where the *P*-value < 0.05.

Using the P450 sequences from the *Drosophila*, we predicted the target P450 gene of miRNAs using miRNA seed regions binding to the 3′ UTR of mRNAs. We found the 3′ UTR sequence of *Cyp6g1*, *Cyp6g2*, *Cyp6a8*, and *Cyp4g1* contained a target site for *miR-310-3p*, *311-3p*, *312-3p*, *313-3p*, and *92a-3p* (Supplementary Figure S3). The relative expression of corresponding putative P450 targets of the eight miRNAs (Table 1) were estimated using RT-qPCR and correlations made with the expression levels estimated for corresponding miRNAs. Specifically, estimates of RT-qPCR showed that *Cyp6g1* was induced significantly in *Canton-S*, with up-regulation of 6.4-fold at 24 h, 3.2-fold at 48 h, and 4-fold at 72 h after DDT treatment (Figure 3A). The corresponding relative expression level of *Cyp6g2* was also significantly increased 1.8-, 2.11-, and 1.78-fold in *Canton-S* strain respectively at 24, 48, and 72 h after DDT exposure compared to control flies (Figure 3B). The relative expression level of *Cyp6a8* in *Canton-S* was significantly up-regulated 8.0-, 6.1-, and 7.7-fold compared to control flies at 24, 48, and 72 h after DDT exposure (Figure 3C). Additionally, the expression of *Cyp4g1* was significantly up-regulated 2.0- 1.5-, and 1.6-fold in *Canton-S* respectively at 24, 48, and 72 h after DDT exposure (Figure 3D). In general, expression of P450s generally was increased in response to DDT compared to control flies and remained significantly lower in *Canton-S* compared to *91-R* strains across all time points following DDT-exposure (*P*-values < 0.05).

**Table 1:** The potential DDT resistance-related target P450s of the differentially expressed miRNAs.

Target gene^∗^	miRNA	Reference for DDT resistance
*Cyp6g1*	*miR-310-3p*	Daborn et al., 2001
	*miR-311-3p*	
	*miR-312-3p*	
	*miR-313-3p*	
*Cyp6g2*	*miR-310-3p*	Pedra et al., 2004
	*miR-311-3p*	
	*miR-313-3p*	
	*miR-92a-3p*	
*Cyp6a8*	*miR-312-3p*	Pedra et al., 2004
*Cyp4g1*	*miR-312-3p*	Gellatly et al., 2015
	*miR-92a-3p*	


*^∗^All target genes were predicted with RNAhybrid (Kruger and Rehmsmeier, 2006), DIANA (Maragkakis et al., 2009), ComiR (Coronnello and Benos, 2013), and miRanda (Enright et al., 2003).*

The levels of expression among these putative target P450s for *Canton-S* exhibited opposite expression patterns with their corresponding miRNAs at the same time points after DDT exposure. For example, the relative expression level of *Cyp6a8* in *Canton-S* was significantly up-regulated compared to control flies at 24, 48, and 72 h after DDT exposure. At the same time points, its corresponding miRNA, *miR-312-3p*, showed significant down-regulation in response to DDT exposure. Similarly, *Cyp4g1* was predicted as a potential target for *miR-312-3p* and *miR-92a-3p*. The expressions of *miR-312-3p* and *miR-92a-3p* were significantly down-regulated at 24, 48, and 72 h in response to DDT exposure in *Canton-S*, while the expression of their corresponding target, *Cyp4g1*, was significantly up-regulated at the same time points after DDT exposure. *Cyp6g2* was predicted as a putative target for *miR-310-3p*, *miR-311-3p*, *miR-313-3p*, and *miR-92a-3p* and the expression of *Cyp6g2* was significantly up-regulated at 24, 48, and 72 h after DDT exposure, while the expressions of corresponding miRNAs were significantly down-regulated at 24 and 48 h for *miR-310-3p* and *miR-313-3p*, at 12, 24, and 48 h for *miR-311-3p*, and at 24, 48, and 72 h for *miR-92a-3p* in response to DDT exposure in *Canton-S*. Relative expression level of *Cyp6g1* in *Canton-S* as well was significantly up-regulated compared to control flies at 24, 48, and 72 h after DDT exposure, while, at the same time points, the putative corresponding regulating miRNA, the *miR-310-313* cluster, showed significant changes that were inversely related. Overall, although the correlation between miRNAs and their target mRNA expressions was not statistically significant (except a pair of *miR-92a* and *Cyp6g2* : *P-value* = 0.014; Supplementary Table S4), mRNA expression of target P450s showed a negative relationship with the expression of their corresponding miRNAs at the same time points after DDT exposure.

## Discussion

High-throughput small RNA (sRNA) sequencing from the DDT-resistant *Drosophila* strain, *91-R*, previously revealed that 10 miRNAs were constitutively differentially expressed when compared with the susceptible control strain, *91-C*, and predicted that these miRNAs putatively target a number of P450 gene transcripts (Seong et al., 2018b). The present study adds to this knowledge by showing that among these 10 differentially expressed miRNAs, *miR-310-3p*, *miR-311-3p*, *miR-312-3p*, *miR-313-3p*, and *miR-92a-3p* have significantly decreased levels of expression following DDT exposure at different time points in the wild-type DDT susceptible strain *Canton-S*. Furthermore, we showed that the expression of the putative target P450 targets, *Cyp6a8*, *Cyp6g1*, *Cyp6g2*, and *Cyp4g1*, have corresponding significantly increased levels of expression in *Canton-S* following DDT exposure, which is an inverse pattern of response compared to their predicted targeting miRNAs at some time points. Interestingly, our results demonstrate that significant differences in expression among the six miRNAs (*miR-310-3p*, *miR-311-3p*, *miR-312-3p*, *miR-313-3p*, *miR-92a-3p*, and *miR-986-5p*) between susceptible *Canton-S* and highly resistant *91-R* strains are inducible following DDT exposures, as opposed to being constitutive. Specifically, this study showed that the exposure to DDT has no significant impact on miRNA expression levels in *91-R*, whereas the same treatments led to a significant induction of down-regulation in miRNA expression in *Canton-S* at different time points (*P*-values < 0.05).

It was previously shown that miRNA levels are induced or repressed following animal exposures to chemical insecticides. For example, *miR-21*, *miR-221*, and *miR-222* expression levels were estimated to be 2–3 times lower in DDT-treated rat liver compared to untreated controls (Gulyaeva et al., 2016). Recently, analogous levels of differential miRNA expression patterns were associated with insecticide resistance or were shown to be induced following exposure to insecticides. Specifically, the expression of *miR-216b* and *miR-499* were down-regulated in *Danio rerio* following exposure to the phenylpyrazole insecticide, fipronil (Zhou et al., 2016). Comparative analyses have estimated significant levels of differential expression in 33 miRNAs when comparing imidacloprid-exposed and non-exposed control groups of the Colorado potato beetle, *Leptinotarsa decemlineata* (Morin et al., 2017). Additionally, following different concentrations and durations of exposure of *P. xylostella* to the ryanoid insecticide, chlorantraniliprole, several miRNAs were significantly changed in abundance (Etebari et al., 2018). These prior, and our current, results indicate that the inducible expression level changes of miRNAs in response to insecticides may play a potential role in arthropod responses to chemical insecticides exposures and that these miRNAs might be involved in the regulating the expression of multiple target genes facilitating survival in insect species.

In this study, five miRNAs were down-regulated in response to DDT exposure in *Canton-S* (*miR-310-3p*, *miR-311-3p*, *miR-312-3p*, *miR-313-3p*, and *miR-92a-3p*) belonging to the miR-310 family, which are clustered within the *Drosophila* genome. Although *miR-313-3p* is derived from the same precursor as *-5p*, the latter was not differentially expressed. This is consistent with recent evidence that despite these two miRNAs being derived from as a single precursor, *miR-313-3p* and *-5p* can show independent regulation (Zhao et al., 2018). Among these five miRNAs, the *miR-310-313* cluster is required for regulating neurotransmitter release in motor neurons during synaptic growth in *Drosophila* larval development (Tsurudome et al., 2010). Additionally, the miR-310 family can down-regulate the *escargot* (*esg*) gene that is involved in nicotine sensitivity in *Drosophila* (Sanchez-Diaz et al., 2015). Sanchez-Diaz et al. (2015) observed that the over-expression of *miR-310-313* led to a significant decrease in the *esg* transcription levels, thereby disrupting the function of sensory organs associated with chemical perception as well as cuticle development. The current research validated that the significant down-regulation of *miR-310-313* was induced in wild-type DDT susceptible strain *Canton-S* following DDT exposure, suggesting the possibility that the *miR-310-313* cluster may be regulated to mediate functional targets involved in neurotransmitter release in response to neurotoxicity of DDT. Interestingly, Seong et al. (2017) also demonstrated that differences in expression of transcripts involved in neurologic function are fixed between *91-R* and its susceptible control strain, *91-C*. This suggestively hints that a connection may lay between the differential expression of miR-310 family and potential differences in response of the nervous system to DDT exposures, but such a hypothesis still requires experimental testing.

Several P450 genes, including *Cyp6a8*, *Cyp6g1*, *Cyp6g2*, and *Cyp4g1* were previously associated with metabolic resistance against DDT in *Drosophila* (Seong et al., 2018a). For example, *Cyp6a8*, *Cyp6g1*, and *Cyp6g2* are constitutively up-regulated in DDT-resistant *91-R* strain compared to susceptible counterparts (Pedra et al., 2004; Seong et al., 2017). Furthermore, it was previously shown that *Cyp4g1* is constitutively up-regulated in the DDT-resistant *91-R* strain compared to the insecticide-susceptible *Canton-S* in untreated conditions (Gellatly et al., 2015) and *Cyp4g1* is located within a genome region previously defined as having undergone a selective sweep that reduced nucleotide diversities in *91-R* as compared to the susceptible counterpart *91-C*, and thus was putatively associated with the evolution of DDT resistance (Steele et al., 2015). Since *Cyp4g1* is involved in endogenous metabolism that is responsible for the decarbonylation of long chain aldehydes to form cuticular hydrocarbons (Qiu et al., 2012), it could be possible that up-regulation functions to reduce DDT penetration through the cuticle. Findings from this current study further demonstrate that the expression of *Cyp6a8*, *Cyp6g1*, *Cyp6g2*, and *Cyp4g1* are significantly induced after DDT exposure in susceptible *Canton-S*, which compares to their constitutive and higher relative expression in *91-R*. Interestingly, the increased expression of the targeted P450s in *Canton-S* is accompanied by the significant down-regulation of their putative targeting *miR-310-3p*, *miR-311-3p*, *miR-312-3p*, *miR-313-3p*, and *miR-92a-3p*.

Prior research has implicated miRNAs in the post-transcriptional regulation of P450 expression levels through the binding of the 3′-UTRs of transcripts for the latter. For example, the expression of P450s *Cyp9j35*, *Cyp325bg3*, and *Cyp6n23* is controlled by *miR-2∼13∼71* cluster and *miR-285*, and this regulatory network is directly involved in pyrethroid insecticide resistance in *Culex pipiens* (Tian et al., 2016; Guo et al., 2017). Evidence from the current study demonstrate that the expression level of P450 genes and corresponding putative targeting miRNAs, *miR-310-3p*, *miR-311-3p*, *miR-312-3p*, *miR-313-3p* and *miR-92a-3p*, showed an inverse relationship at 24, 48, and 72 h after DDT exposure in the susceptible strain *Canton-S*. These associative data may suggest that the down-regulation of miRNAs could be involved in the regulation of P450 expression levels in *Canton-S.* However, these interactions need to be validated by functional experiments in order to confirm predicted interactions between miRNAs and putative target genes *in vitro* or *in vivo*. Therefore, the potential that these miRNAs are involved in post-transcriptional regulation of P450s, or modulate associated biochemical pathways which influence DDT resistance phenotypes remains unknown. Regardless, the inducible expression of miRNAs and their corresponding putatively targeted P450s in response to DDT exposure enticingly suggests the potential role of *Drosophila* miRNAs in coordinating detoxification responses during the DDT exposure or (alternatively) could point to their involvement in a general response to cellular stress pathway.

Temporal differences in the responses of miRNA expression following DDT exposure were predicted between *91-R* and *Canton-S* strains. Specifically, some changes of miRNAs expression were transient in *Canton-S* in response to a low dose of DDT, possibly suggesting that *miR-310-3p*, *miR-311-3p*, *miR-312-3p*, *miR-313-3p*, and *miR-92a-3p* may form a response network that is sensitive to DDT exposure or general cellular stress. The involvement of these changes in the response of susceptible *Drosophila* to DDT detoxification remains undetermined, but might involve mechanisms for short-term cellular clearance of xenobiotics or responses to cellular stress conditions prior to the reestablishment of homeostasis. As stated above, the temporal expression level of these same miRNAs remains relatively unchanged at low levels in *91-R* following the same DDT exposures. These results could suggest that reduced miRNA expression, and corresponding putative reduced levels of post-transcriptional repression of target P450s, may represent adaptive changes in response to multigenerational neurotoxic DDT selection in *91-R*. Specifically, the selection for mutations in miRNA regulation that impact the degree to which miRNAs are induced following exposure to DDT may have become fixed in *91-R* and resulted in the observed fixed differences in expression. Therefore, the constitutive down-regulation of multiple miRNAs and the general lack of induction following DDT in *91-R* could be indicative of adaptive changes that modify cellular responses via underlying regulatory mechanisms of miRNA expression. Previous work analogously reported that inducible expression of detoxification genes, including P450s, GSTs or esterase genes, are not inducible in response to insecticide exposures, even though these genes have roles in insecticide resistance and metabolism in *Drosophila* (Willoughby et al., 2006). The specific mutations that give rise to these regulatory changes in miRNA expression in *91-R* remain unknown, but undoubtedly will be the focus of future research.

## Conclusion

Insects have metabolic detoxification systems that allow transient changes in response to variations in environmental conditions, including responses to chemical insecticide exposure. Furthermore, selection for mutations that enhance cellular detoxification of xenobiotics are known within insecticide-resistant phenotypes of various arthropod species. A number of studies indicate that both constitutively increased expression and induction of detoxification genes such as P450s, GSTs, and esterases in insects are linked or associated with insecticide resistance traits and the ability of arthropods to adapt to a changing environment (Russell et al., 2011; Ffrench-Constant, 2013). As such, miRNAs are now known as mediators of detoxification gene expression levels via post-transcriptional control, which has led to their candidacy as potential regulators of detoxification pathway (Wang and Cui, 2012; Yu and Cho, 2015). By integrating the expression of both differentially expressed miRNAs in conjunction with their predicted target P450s, this study identified an inverse pattern between the two in the DDT susceptible strain, *Canton-S*. In contrast, the relative level of expression among miRNAs remained lower and the P450 genes constitutively higher in *91-R* compared to *Canton-S*.

As such, we demonstrated that inducible changes in miRNA in response to low-level DDT exposures may impact temporal expression levels of P450s in *Canton-S*, thus highlighting the importance of transient changes in expression levels in the response to cellular stress. Furthermore, this study showed the likely influence of adaptive changes in miRNA expression in response to DDT selection and the evolution of the resistant phenotype in *91-R*. This study provides important information regarding the changes in miRNA expression levels and the regulation of detoxification genes in short term responses to low-level DDT exposures, as well as within adaptive phenotypes that are resistant to high levels of DDT phenotypes, and increases scientific understanding of potential involvement of miRNAs in the adaption of stress-response pathways in DDT resistant strains of *Drosophila.*

## Author Contributions

KS performed most of the experiments with the help of BC and BP. KS analyzed the results and wrote the manuscript assisted by BC and BP. All authors reviewed and approved the manuscript.

## Conflict of Interest Statement

The authors declare that the research was conducted in the absence of any commercial or financial relationships that could be construed as a potential conflict of interest.
