# Supplementary material for: Impacts of Sub-lethal DDT Exposures on microRNA and Putative Target Transcript Expression in DDT Resistant and Susceptible Drosophila melanogaster Strains
Source: Front Genet. 2019 Feb 5;10:45. doi: 10.3389/fgene.2019.00045 (PMC6370691; doi:10.3389/fgene.2019.00045)
Supplement: Supplementary file 1 [file Data_Sheet_1.docx]

Supplementary Material

**Impacts of sub-lethal DDT exposures on microRNA and putative target transcript expression in DDT resistant and susceptible *Drosophila melanogaster* strains**

**Keon Mook Seong^1*^, Brad S. Coates^2^, Barry R. Pittendrigh^1^**

^1^ Department of Entomology, Michigan State University, East Lansing, MI, USA

^2^ USDA-ARS, Corn Insects & Crop Genetics Research Unit, Ames, IA USA

*** Correspondence:** Keon Mook Seong: seongkeo@msu.edu

**Supplementary Figure S1.** The expression of *miR-286-3p* family in DDT-resistant *91-R* and control

*Canton-S* strains following DDT exposure. The induction or repression level of expression were analyzed 12, 24, 48, and 72hr after DDT exposure. The relative levels of gene expression shown along the Y axis represent the ratio of the gene expression in each treatment in comparison with that in acetone treated *Canton-S* strain. The experiments were repeated three times. The vertical bars indicate standard error of the mean (SEM). Different letters on the bars indicate that the means are significantly different throughout the strains where the *P*-value < 0.05.

**Supplementary Figure S2.** The expression of *miR-4919-3p* in DDT-resistant *91-R* and control *Canton-S* strains following DDT exposure. The induction or repression level of expression were analyzed 12, 24, 48, and 72hr after DDT exposure. The relative levels of gene expression shown along the Y axis represent the ratio of the gene expression in each treatment in comparison with that in acetone treated *Canton-S* strain. The experiments were repeated three times. The vertical bars indicate standard error of the mean (SEM). Different letters on the bars indicate that the means are significantly different throughout the strains where the *P*-value < 0.05.

**Supplementary Figure S3.** Potential miRNA target sites of *miR-310-3p*, *311-3p*, *312-3p*, *313-3p*, and *92a-3p* in the 3′ -UTR of the P450s as detected by RNAhybrid and miRanda. Seed sequence of the miRNAs and their putative binding sites in the 3′ -UTR are indicated by red box.

**Supplementary Table S1.** The list of RT-qPCR primers.

| **miRNA or gene** | **Forward primers** | **Reverse primers** | **Amplification Efficiency ᵃ** |
| --- | --- | --- | --- |
| *miR-310-3p* | UAUUGCACACUUCCCGGCCUUU | Universal primer (supplied from miScript SYBR^®^ Green PCR Kit) | 1.97 |
| *miR-311-3p* | UAUUGCACAUUCACCGGCCUGA | Universal primer (supplied from miScript SYBR^®^ Green PCR Kit) | 1.97 |
| *miR-312-3p* | UAUUGCACUUGAGACGGCCUGA | Universal primer (supplied from miScript SYBR^®^ Green PCR Kit) | 1.86 |
| *miR-313-3p* | UAUUGCACUUUUCACAGCCCGA | Universal primer (supplied from miScript SYBR^®^ Green PCR Kit) | 1.85 |
| *miR-986-5p* | UCUCGAAUAGCGUUGUGACUGA | Universal primer (supplied from miScript SYBR^®^ Green PCR Kit) | 1.85 |
| *miR-286-3p* | UGACUAGACCGAACACUCGUGCU | Universal primer (supplied from miScript SYBR^®^ Green PCR Kit) | 2.1 |
| *miR-92a-3p* | CAUUGCACUUGUCCCGGCCUAU | Universal primer (supplied from miScript SYBR^®^ Green PCR Kit) | 2.1 |
| *miR-4919-3p* | UAAUCCCUGAACGACUUGCAG | Universal primer (supplied from miScript SYBR^®^ Green PCR Kit) | 1.93 |
| *5S rRNA* | CGACCATACCACGCTGAATA | Universal primer (supplied from miScript SYBR^®^ Green PCR Kit) | 2.2 |
| *U6 snRNA* | TGGCCCCTGCGCAAGGATG | Universal primer (supplied from miScript SYBR^®^ Green PCR Kit) | 1.91 |
| *rp49* | CGGATCGATATGCTAAGCTGT | GCGCTTGTTCGATCCGTA | 1.95 |
| *Cyp4g1* | CTACTCCTTCATTCCCTTTAGC | TCGGTGGAGTGGACAATA | 1.98 |
| *Cyp6a8* | GCACCGCATATTCAGGAATTC | GACCACATCGCCATTCTCTAG | 1.92 |
| *Cyp6g1* | TGTCTTTTGCCCTGTACGAG | CAGGGACTGGATTTTCTCGTAG | 2.1 |
| *Cyp6g2* | AACTATGTGATGTCGGAGCG | CCTTGATACCCGATGCCTTC | 1.95 |

^ᵃ^Primer efficiencies were determined from a serial dilution of target cDNA using the formula: E = 10^(-1/slope)^.

**Supplementary Table S2.** ANOVA test for the miRNA expression within acetone treated control flies from *91-R* and *Canton-S* strains across different time points.

| **Strain** | **miRNA** | ***df*** | **F** | ***P*-value** |
| --- | --- | --- | --- | --- |
| *91-R* | *miR-310-3p* | 11 | 1.34 | 0.329 |
| *Canton-S* |  | 11 | 2.44 | 0.139 |
| *91-R* | *miR-311-3p* | 11 | 1.34 | 0.329 |
| *Canton-S* |  | 11 | 1.01 | 0.437 |
| *91-R* | *miR-312-3p* | 11 | 1.47 | 0.293 |
| *Canton-S* |  | 11 | 0.36 | 0.784 |
| *91-R* | *miR-313-3p* | 11 | 0.94 | 0.467 |
| *Canton-S* |  | 11 | 1.99 | 0.193 |
| *91-R* | *miR-92a-3p* | 11 | 1.66 | 0.252 |
| *Canton-S* |  | 11 | 2.05 | 0.186 |
| *91-R* | *miR-286-3p* | 11 | 1.07 | 0.415 |
| *Canton-S* |  | 11 | 1.01 | 0.435 |
| *91-R* | *miR-4919-3p* | 11 | 0.56 | 0.654 |
| *Canton-S* |  | 11 | 0.27 | 0.843 |
| *91-R* | *miR-986-5p* | 11 | 2.27 | 0.156 |
| *Canton-S* |  | 11 | 0.79 | 0.533 |

**Supplementary Table S3.** Two way-ANOVA test for the miRNA and P450 expressions in response to DDT exposure across two strains at different time points.

| **Gene** | **Source** | **DF** | **Mean squares** | **F** | **Pr > F** |
| --- | --- | --- | --- | --- | --- |
| *miR-310-3p*_12h | strains | 1 | 0.326 | 13.304 | **0.007** |
|  | treatment | 1 | 0.083 | 3.400 | 0.102 |
|  | strains*treatment | 1 | 0.024 | 0.992 | **0.035** |
| *miR-310-3p*_24h | strains | 1 | 0.149 | 10.709 | **0.011** |
|  | treatment | 1 | 0.206 | 14.764 | **0.005** |
|  | strains*treatment | 1 | 0.199 | 14.290 | **0.005** |
| *miR-310-3p*_48h | strains | 1 | 0.130 | 6.905 | **0.030** |
|  | treatment | 1 | 0.364 | 19.302 | **0.002** |
|  | strains*treatment | 1 | 0.175 | 9.291 | **0.016** |
| *miR-310-3p*_72h | strains | 1 | 0.488 | 23.426 | **0.001** |
|  | treatment | 1 | 0.032 | 1.538 | 0.250 |
|  | strains*treatment | 1 | 0.178 | 8.526 | **0.019** |
| *miR-311-3p*_12h | strains | 1 | 0.513 | 47.641 | **0.000** |
|  | treatment | 1 | 0.282 | 26.225 | **0.001** |
|  | strains*treatment | 1 | 0.032 | 2.978 | **0.123** |
| *miR-311-3p*_24h | strains | 1 | 0.946 | 113.797 | **< 0.0001** |
|  | treatment | 1 | 0.357 | 42.935 | **0.002** |
|  | strains*treatment | 1 | 0.298 | 35.793 | **0.003** |
| *miR-311-3p*_48h | strains | 1 | 0.371 | 51.589 | **< 0.0001** |
|  | treatment | 1 | 0.279 | 38.805 | **0.003** |
|  | strains*treatment | 1 | 0.576 | 80.149 | **< 0.0001** |
| *miR-311-3p*_72h | strains | 1 | 0.755 | 89.971 | **< 0.0001** |
|  | treatment | 1 | 0.092 | 10.948 | **0.011** |
|  | strains*treatment | 1 | 0.066 | 7.866 | **0.023** |
| *miR-312-3p*_12h | strains | 1 | 1.056 | 135.547 | **< 0.0001** |
|  | treatment | 1 | 0.003 | 0.347 | 0.572 |
|  | strains*treatment | 1 | 0.003 | 0.347 | 0.572 |
| *miR-312-3p*_24h | strains | 1 | 0.504 | 66.574 | **< 0.0001** |
|  | treatment | 1 | 0.354 | 46.684 | **0.001** |
|  | strains*treatment | 1 | 0.513 | 67.661 | **< 0.0001** |
| *miR-312-3p*_48h | strains | 1 | 0.205 | 25.050 | **0.001** |
|  | treatment | 1 | 0.317 | 38.643 | **0.003** |
|  | strains*treatment | 1 | 0.639 | 77.977 | **< 0.0001** |
| *miR-312-3p*_72h | strains | 1 | 0.180 | 18.438 | **0.003** |
|  | treatment | 1 | 0.185 | 18.943 | **0.002** |
|  | strains*treatment | 1 | 0.190 | 19.455 | **0.002** |
| *miR-313-3p*_12h | strains | 1 | 0.354 | 101.522 | **< 0.0001** |
|  | treatment | 1 | 0.141 | 40.431 | **0.002** |
|  | strains*treatment | 1 | 0.018 | 5.062 | 0.055 |
| *miR-313-3p*_24h | strains | 1 | 0.500 | 48.761 | **0.000** |
|  | treatment | 1 | 0.304 | 29.635 | **0.001** |
|  | strains*treatment | 1 | 0.452 | 44.102 | **0.002** |
| *miR-313-3p*_48h | strains | 1 | 0.247 | 28.722 | **0.001** |
|  | treatment | 1 | 0.183 | 21.266 | **0.002** |
|  | strains*treatment | 1 | 0.333 | 38.835 | **0.003** |
| *miR-313-3p*_72h | strains | 1 | 0.301 | 45.069 | **0.002** |
|  | treatment | 1 | 0.320 | 47.960 | **0.001** |
|  | strains*treatment | 1 | 0.071 | 10.567 | **0.012** |
| *miR-92a-3p*_12h | strains | 1 | 0.791 | 91.040 | **< 0.0001** |
|  | treatment | 1 | 0.101 | 11.612 | **0.009** |
|  | strains*treatment | 1 | 0.101 | 11.612 | **0.009** |
| *miR-92a-3p*_24h | strains | 1 | 0.472 | 56.986 | **< 0.0001** |
|  | treatment | 1 | 0.276 | 33.324 | **0.004** |
|  | strains*treatment | 1 | 0.137 | 16.483 | **0.004** |
| *miR-92a-3p*_48h | strains | 1 | 0.195 | 32.832 | **0.004** |
|  | treatment | 1 | 0.190 | 31.979 | **0.005** |
|  | strains*treatment | 1 | 0.407 | 68.501 | **< 0.0001** |
| *miR-92a-3p*_72h | strains | 1 | 0.141 | 17.175 | **0.003** |
|  | treatment | 1 | 0.213 | 26.016 | **0.001** |
|  | strains*treatment | 1 | 0.307 | 37.463 | **0.003** |
| *miR-986-5p*_12h | strains | 1 | 111.082 | 909.822 | **< 0.0001** |
|  | treatment | 1 | 3.956 | 32.402 | **0.005** |
|  | strains*treatment | 1 | 3.532 | 28.926 | **0.001** |
| *miR-986-5p*_24h | strains | 1 | 50.021 | 104.029 | **< 0.0001** |
|  | treatment | 1 | 1.841 | 3.828 | 0.086 |
|  | strains*treatment | 1 | 6.021 | 12.522 | **0.008** |
| *miR-986-5p*_48h | strains | 1 | 68.689 | 142.703 | **< 0.0001** |
|  | treatment | 1 | 0.103 | 0.213 | 0.656 |
|  | strains*treatment | 1 | 1.015 | 2.109 | 0.185 |
| *miR-986-5p*_72h | strains | 1 | 79.156 | 209.297 | **< 0.0001** |
|  | treatment | 1 | 0.015 | 0.039 | 0.849 |
|  | strains*treatment | 1 | 0.028 | 0.074 | 0.792 |
| *Cyp6g1*_12h | strains | 1 | 4786.410 | 565.716 | **< 0.0001** |
|  | treatment | 1 | 1.570 | 0.186 | 0.678 |
|  | strains*treatment | 1 | 0.230 | 0.027 | 0.873 |
| *Cyp6g1*_24h | strains | 1 | 7203.000 | 310.887 | **< 0.0001** |
|  | treatment | 1 | 481.333 | 20.775 | **0.002** |
|  | strains*treatment | 1 | 33.333 | 1.439 | 0.265 |
| *Cyp6g1*_48h | strains | 1 | 10016.741 | 882.791 | **< 0.0001** |
|  | treatment | 1 | 1808.108 | 159.351 | **< 0.0001** |
|  | strains*treatment | 1 | 1174.141 | 103.479 | **< 0.0001** |
| *Cyp6g1*_72h | strains | 1 | 5590.083 | 698.542 | **< 0.0001** |
|  | treatment | 1 | 80.083 | 10.007 | **0.013** |
|  | strains*treatment | 1 | 30.083 | 3.759 | 0.088 |
| *Cyp6g2*_12h | strains | 1 | 86.403 | 249.841 | **< 0.0001** |
|  | treatment | 1 | 0.003 | 0.010 | 0.924 |
|  | strains*treatment | 1 | 0.163 | 0.472 | 0.511 |
| *Cyp6g2*_24h | strains | 1 | 126.750 | 192.288 | **< 0.0001** |
|  | treatment | 1 | 26.403 | 40.056 | **0.000** |
|  | strains*treatment | 1 | 6.453 | 9.790 | **0.014** |
| *Cyp6g2*_48h | strains | 1 | 349.920 | 198.161 | **< 0.0001** |
|  | treatment | 1 | 152.653 | 86.448 | **< 0.0001** |
|  | strains*treatment | 1 | 52.920 | 29.969 | **0.001** |
| *Cyp6g2*_72h | strains | 1 | 104.430 | 132.891 | **< 0.0001** |
|  | treatment | 1 | 5.070 | 6.452 | **0.035** |
|  | strains*treatment | 1 | 3.413 | 4.344 | 0.071 |
| *Cyp6a8*_12h | strains | 1 | 27018.030 | 680.912 | **< 0.0001** |
|  | treatment | 1 | 5834.430 | 147.040 | **< 0.0001** |
|  | strains*treatment | 1 | 5435.763 | 136.993 | **< 0.0001** |
| *Cyp6a8*_24h | strains | 1 | 5555.603 | 1176.827 | **< 0.0001** |
|  | treatment | 1 | 71.053 | 15.051 | **0.005** |
|  | strains*treatment | 1 | 31.363 | 6.644 | **0.033** |
| *Cyp6a8*_48h | strains | 1 | 5423.001 | 809.302 | **< 0.0001** |
|  | treatment | 1 | 58.521 | 8.733 | **0.018** |
|  | strains*treatment | 1 | 50.021 | 7.465 | **0.026** |
| *Cyp6a8*_72h | strains | 1 | 6566.041 | 693.474 | **< 0.0001** |
|  | treatment | 1 | 31.041 | 3.278 | 0.108 |
|  | strains*treatment | 1 | 86.941 | 9.182 | **0.016** |
| *Cyp4g1*_12h | strains | 1 | 3.308 | 61.062 | **< 0.0001** |
|  | treatment | 1 | 1.841 | 33.985 | **0.004** |
|  | strains*treatment | 1 | 1.841 | 33.985 | **0.004** |
| *Cyp4g1*_24h | strains | 1 | 2.430 | 138.857 | **< 0.0001** |
|  | treatment | 1 | 3.853 | 220.190 | **< 0.0001** |
|  | strains*treatment | 1 | 0.003 | 0.190 | 0.674 |
| *Cyp4g1*_48h | strains | 1 | 3.203 | 45.762 | **0.001** |
|  | treatment | 1 | 2.613 | 37.333 | **0.003** |
|  | strains*treatment | 1 | 0.013 | 0.190 | 0.741 |
| *Cyp4g1*_72h | strains | 1 | 0.563 | 8.779 | **0.018** |
|  | treatment | 1 | 0.480 | 7.481 | **0.026** |
|  | strains*treatment | 1 | 0.653 | 10.182 | **0.013** |

**Supplementary Table S4. Correlation analysis of expression of differentially expressed miRNAs and their targets P450s.**

| **Target gene** | **miRNA** | **Correlation coefficient (*r*)** | **95% Confidence interval** | ***P*-value** |
| --- | --- | --- | --- | --- |
| *Cyp6g1* | *miR-310-3p* | -0.348 | -0.982 ~ 0.921 | 0.652 |
| *Cyp6g1* | *miR-311-3p* | -0.523 | -0.988 ~ 0.881 | 0.477 |
| *Cyp6g1* | *miR-312-3p* | -0.742 | -0.994 ~ 0.764 | 0.258 |
| *Cyp6g1* | *miR-313-3p* | -0.379 | -0.982 ~ 0.916 | 0.621 |
| *Cyp6g2* | *miR-310-3p* | -0.487 | -0.986 ~ 0.891 | 0.513 |
| *Cyp6g2* | *miR-311-3p* | -0.688 | -0.993 ~ 0.806 | 0.312 |
| *Cyp6g2* | *miR-313-3p* | -0.254 | -0.977 ~ 0.935 | 0.746 |
| ***Cyp6g2*** | ***miR-92a-3p*** | **-0.986** | **-1 ~ -0.487** | **0.014** |
| *Cyp6a8* | *miR-313-3p* | -0.08 | -0.967 ~ 0.955 | 0.922 |
| *Cyp4g1* | *miR-312-3p* | -0.767 | -0.995 ~ 0.738 | 0.233 |
| *Cyp4g1* | *miR-92a-3p* | -0.651 | -0.992 ~ 0.829 | 0.349 |
